# Food purchase patterns: empirical identification and analysis of their association with diet quality, socio-economic factors, and attitudes

**DOI:** 10.1186/s12937-017-0292-z

**Published:** 2017-10-12

**Authors:** Silke Thiele, Jonas Peltner, Almut Richter, Gert B. M. Mensink

**Affiliations:** 1 Department of Food Economics and Consumption Studies, University of Kiel, Olshausenstrasse 40, D-24098 Kiel, Germany; 2 ife, Institute of Food Economics, Fraunhoferstrasse 13, D-24118 Kiel, Germany; 3 Department of Epidemiology and Health Monitoring, Robert Koch Institute Berlin, Post box 65 02 61, D-13302 Berlin, Germany

**Keywords:** Food purchase patterns, Diet quality, Socio-economic factors, Attitudes

## Abstract

**Background:**

Empirically derived food purchase patterns provide information about which combinations of foods were purchased from households. The objective of this study was to identify what kinds of patterns exist, which level of diet quality they represent and which factors are associated with the patterns.

**Methods:**

The study made use of representative German consumption data in which approximately 12 million food purchases from 13,125 households are recorded. In accordance with healthy diet criteria the food purchases were assigned to 18 food groups of the German Food Pyramid. Based on these groups a factor analysis with a principal component technique was applied to identify food patterns. For these patterns nutrient and energy densities were examined. Using regression analysis, associations between pattern scores and socio-economic as well as attitude variables, reflecting personal statements about healthy eating, were analyzed.

**Results:**

In total, three food purchase patterns could be identified: a natural, a processed and a traditional one. The first one was characterized by a higher purchasing of natural foods, the second by an increased purchasing of processed foods and the third by a meat-oriented diet. In each pattern there were specific diet quality criteria that could be improved whereas others were in line with actual dietary guidelines. In addition to socio-demographic factors, attitudes were significantly associated with the purchase patterns.

**Conclusions:**

The findings of this study are interesting from a public health perspective, as it can be assumed that measures focusing on specific aspects of diet quality are more promising than general ones. However, it is a major challenge to identify the population groups with their specific needs of improvement. As the patterns were associated with both socio-economic and attitude variables these grouping criteria could be used to define target groups.

## Background

An unfavorable diet quality is a big public health issue in many industrialized countries. According to the latest diet report for Germany [1] a poor diet quality in combination with lack of exercise is responsible for the increasing number of overweight people in Germany which is currently 59% for men and 37% for women. In addition, a poor diet quality is directly attributed to the development of diet-related-diseases such as diabetes and cardiovascular diseases and stroke. In particular, the prevalence of diabetes type II has increased and this is only partially explained by demographic ageing but mainly by unfavorable lifestyle factors [2, 3]. Experts constantly emphasize that a balanced diet, avoidance of overweight, and an increased physical activity could help to reduce the development of diet-related-diseases [3, 4].

In order to take targeted actions against further increases of diet-related-diseases, it is essential to continuously improve the information base concerning population’s diet quality and its associated factors. To get information about the diet quality, the international literature increasingly refers to dietary patterns [5, 6]. When the patterns are empirically derived they provide information about which foods are often combined by the consumers [7, 8]. Because not individual foods but the combination of foods determines people’s supply of energy and nutrients a dietary pattern approach is particularly appropriate in describing diet quality [5].

So far, only some studies have been carried out for Germany dealing with the identification of dietary patterns. Two of these studies examined dietary patterns among adolescents [9, 10] all others were focused on adults [11–19]. Basically, two patterns could be distinguished: a healthy pattern which was characterized by a frequent purchasing of fruits, vegetables and whole meal products [9–16] and a “western” or “processed” pattern characterized by a higher purchasing of red meat and highly processed foods [9, 15–18]. In some studies, patterns with a high purchasing of alcohol [11] or traditional foods [9, 13, 19] were found. In general, the patterns were used to analyze associations with diet quality [9–13], cardiovascular diseases [15–19], weight changes [14], and socio-economic factors [9, 11, 15, 16].

All mentioned analyses used individual food intake data for the identification of dietary patterns. Although this kind of data is considered particularly suitable in measuring dietary habits, they also have some disadvantages: Firstly, the data are often based on food-frequency-questionnaires, so that food quantities are not precisely recorded and, secondly, they have to deal with the problem of over- and underreporting. As both aspects could lead to biased results, it is highly interesting to conduct a similar analysis using food purchase data in which the mentioned problems are less relevant.

The objective of this study was to present new results on food patterns identified on the basis of representative German household purchase data whereby the main focus was on finding out how consumers combine foods according to the health-related value of foods. It is interesting, to know if patterns exist in which some consumer groups combine mainly favorable foods (fruits, vegetables, whole-meal-products) and other groups combine mainly unfavorable foods (sweets, snacks, sausages) or if completely different patterns exist where consumers combine favorable with unfavorable foods. Such consumer groups may have specific aspects in which their diet quality should be improved and therefore they are possibly in need of different public health measures.

This study aimed at identifying food purchase patterns and specific dietary issues associated with them. The results can be used for creating group specific dietary recommendations. In order to identify the household groups with their specific needs of improvement of diet quality, associations with socio-economic characteristics and attitudes were investigated. The attitudes reflect personal opinions and refer to dietary guidelines, supplements, and fortified foods.

## Methods

### Data

This study used a consumer panel dataset collected over the period from January to December 2011 by the German ‘Gesellschaft für Konsumforschung’ (GfK), a market research institution. The data include all food purchases for consumption at home of 13,125 households which are representative for Germany. Households in the panel are recruited by the GfK based on a two-stage quota sample. In the first stage, households were recruited based on quota for geographical areas, age, household size, and nationality. In a second stage, the sample was adjusted using sampling weights, such as: state, size of town, household size, age of the person in charge of the household, number of children in different age groups, and nationality. The quotas were taken from the German micro census, an annual random sample of 1% of the German population. In the micro census the persons are obliged to participate. Households in the panel participate over multiple time periods, when a household leaves the panel a new household is recruited in compliance with the quotas.

The participating households were requested to document all their purchases at least for a 10 month period per year. For this purpose a bar code scanner was provided by the GfK. All articles with bar codes were scanned directly, for those without a printed bar code (e.g., fresh products bought at weekly markets or bakeries), the household received a book with extra bar codes. By this means, a total of 12,408,473 food purchases, including price, quantity, store types and several other information, were collected by the GFK. Once a year, the person responsible for the household’s food purchases was asked to complete a standardized questionnaire to obtain information on general household characteristics such as age, education, income and, furthermore, a Likert-scale questionnaire including statements related to attitudes. On a scale from 1 (strongly disagree) to 5 (strongly agree) the persons were asked to rate statements relating to dietary guidelines (e.g., ‘We pay attention to a food’s sugar content’), and the usage of supplements and fortified foods (e.g., ‘We use vitamin and/or minerals supplements.’). It can be assumed that the attitudes of the person responsible for the household’s food purchases approximately reflect the attitudes of the household as a whole. This assumption can be derived from economic household theory where it is assumed that households are either a unified decision-making unit or they are individual members of multi-person households keeping their own preferences and constraints. In the latter case households have, for example, a social welfare function reflecting a household consensus [20].

Because the household data set contained no details about the nutritional values of foods this information was linked to the data. For this purpose, the German food composition database (Bundeslebensmittelschlüssel Version 3.01, BLS), which gives information on nutritional values for 14,814 foods available in the German market, was used. As the BLS includes information on nutrients for foods with and without inedible kitchen waste it was possible to link the respective form to the purchase data which give information on different forms of processing (e.g., potatoes peeled or unpeeled).

Linking foods to the BLS resulted in a reduction of the number of products from 6033 to 1954. The main reason for this reduction is the extremely in-depth documentation of the purchase data whereas the nutrient database is less detailed. In the purchase data, for example, every flavor and fat content classification is documented. In the nutrient composition data, in contrast, flavors and fat content classifications are grouped together to broader groups because nutrient analyses were not carried out in such detail. For example, whereas the GfK distinguishes between 168 flavors of yoghurts, they are classified into four flavor groups captured in the BLS (fruit, cereal, natural, soy).

### Classifying food groups

**Fig. 1:**
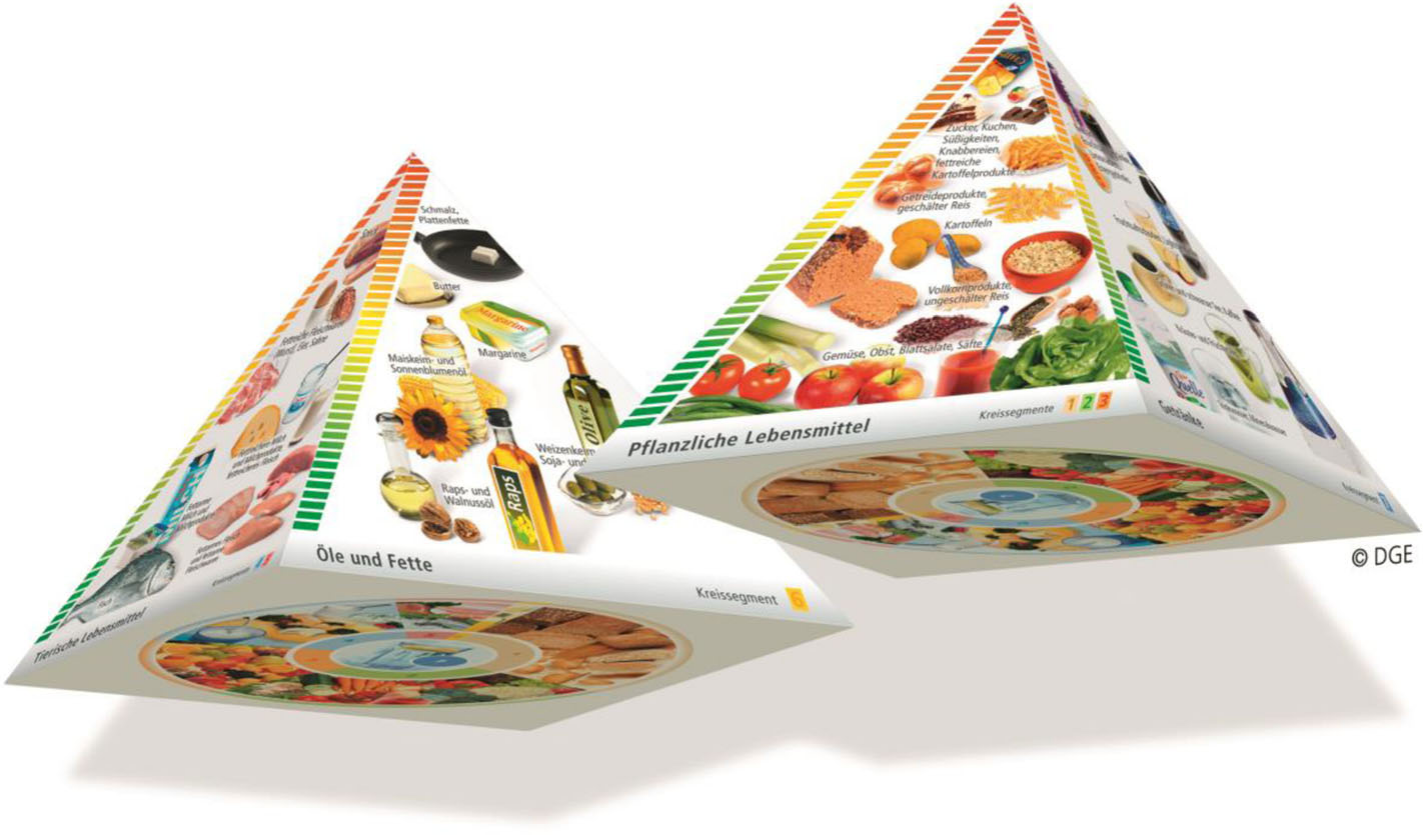
The German Food Guide Pyramid

For the identification of food purchase patterns we applied a factor analysis. For this analysis we aggregated the 1954 food items to food groups. Beverages were not included in this study, because they were not fully available in our data set. As this study was mainly interested in getting deeper insights into how people combine foods according to their health-related evaluation we made use of the food classification scheme of the German Food Guide Pyramid. In this pyramid, foods are grouped together based on energy and nutrient densities, fiber, fatty acid composition as well as known preventive effects on the prevalence of chronic diseases [21]. Considering these criteria foods are first classified into the major groups ‘plant foods’, ‘animal foods’ and ‘fats and oils’ and then, each of them is further divided into subgroups. Foods with a higher health-related evaluation where consumption should be stimulated, are located at the bottom of the pyramid (e.g., vegetables, fruits) and foods with a lower health-related evaluation that should be consumed moderately, are placed at the top (e.g., sugar, snacks). Figure 1 shows a picture of the German Food Guide Pyramid. As criteria such as content of saturated fatty acids determine the allocation to the food groups, foods such as sausages and eggs are in the same pyramid group. Fluid milk is not part of the beverages but is grouped into the animal based food group. Fruit and vegetable juices are also not counted as beverages but belong to the fruit group and vegetable group respectively. The hierarchy within the fats and oils group is essentially determined by the fatty acid composition (saturated and unsaturated fatty acids, omega-3 fatty acids), the vitamin E content, and the content of trans-fatty acids. For reasons of clarity, some foods such as brown bread and cream are not depicted in the graphical illustration of the pyramid. However, studies using or describing the German Food Pyramid [22, 23] show that brown bread is in the same pyramid group as potatoes and cream in the same group as eggs and sausages. Out of the 16 food groups listed in the graphical illustration of the pyramid, we formed 18 food groups for our analysis by splitting the group ‘low fat meat (-products) and fish’ and the group ‘fruits and vegetables’ into two groups each. We classified the 1954 foods available in our dataset into these 18 groups. Mixed products such as salami pizza were assigned to the groups according to their food shares. Therefore, common standard recipes were used to determine the proportional composition of each mixed food product. On this basis, up to four main components were selected to divide the product shares into the respective food groups of the pyramid.

### Statistical methods

Based on the 18 food groups a factor analysis using a principal component technique was applied to identify food purchase patterns. To verify the suitability of the 18 food groups for the factor analysis, communalities were calculated. They measure the percent of variance in a given variable explained by all factors jointly and can be interpreted as the reliability of the respective indicator [24]. The number of factors were retained on the basis of the eigenvalue >1.0 criterion and a plot of the eigenvalues. The orthogonal rotation procedure varimax was used to streamline the interpretation of the results. Food groups that are highly correlated form a factor called purchase pattern. The association between the food groups and the factors is shown formally in the equation *z*_*j*_ = *a*_*j*1_^∗^ *p*_1_ + *a*_*j*2_^∗^ *p*_2_ + … + *a*_*jq*_^∗^ *p*_*q*_, where the z-values represent the standardized quantities ($z=\frac{x-\mu }{\sigma }$, whereby x = raw value, μ = mean and σ = standard deviation) of the food groups (j = 1, .., 23) which are a linear combination of the identified factors (*p* = 1, ..., q) and the factor loadings (a = 1, ..., q). The factor loadings a demonstrate how strong the respective factors p are correlated with the standardized food group quantities z. Using standardized quantities is necessary for two reasons: Firstly, because the different food groups are naturally bought in different amounts (e.g., fats and oils are bought in smaller quantities than vegetables) and secondly, because bigger households buy more than smaller households. Because the standardized quantities are dimensionless scores, they allow comparing the differently distributed random variables.

Based on the standardized quantities of each food group, weighted by the loading of the food group, factor scores were calculated according to the approach of Thurstone [25]. Each household received a score for each identified purchase pattern, with a higher score indicating a higher adherence to the respective pattern. These scores were divided into quintiles, higher quintiles indicating a higher adherence to this pattern. For each quintile of purchase pattern scores mean values of the energy and nutrient densities were calculated, and a trend analysis was conducted using ANOVA. Furthermore, Pearson correlation coefficients between purchase pattern scores and energy and nutrient densities were calculated. The selected nutrients were those for which an insufficient supply is noted in Germany [1]. To analyze household characteristics associated with the adherence to the respective purchase pattern, regression analyses were carried out with the factor scores as the dependent variables and several socio-economic and attitude variables as independent variables. We selected those socio-economic factors for which significant associations with food consumption were found in previous studies. These variables include household income [26, 27], number and composition of household members [28, 29], education level of the principal wage earner [26], age of the person mainly responsible for the food purchases in the household [30, 31], and price level at which food purchases were made [32]. Also for attitudes, significant associations with food consumption were previously detected [33, 34]. To check whether the metric independent variables were linear or non-linear associated with the dependent variable, curve transformations were carried out. By means of the r-square the best curve fits were selected for the final results. In the regression model, all independent variables were included in one step into the equation.

Because the attitude variables have an ordinal scale, we included them in the form of dummy variables. The variables were set to one if the person stated to pay high or very high attention to the respective statement, otherwise it was set to zero. The dataset contains three variables that inform about households’ price level when purchasing foods: the average price for all purchased foods, the share of foods bought in discount stores as well as the share of foods bought as retail brands. Because these variables are closely linked, they were combined using a further factor analysis. The results of this analysis are shown in Table 1.

**Table 1:** Factor loadings for the factor describing the price level at which foods were purchased

Item	Price consciousness
Average price of food purchases	−0.747
Proportion of foods purchased in discount stores	0.753
Proportion of foods purchased as retail brands^a^	0.734
Cronbach’s Alpha	0.7

^a^Retail brands (private labels) are usually cheaper than manufacturing brands

For the factor analyses, the internal validities (reliability) of the factors were checked using Cronbach’s alpha, with values of 0.7 and above considered acceptable [35]. Moreover, goodness-of-fit-measures were calculated to test the fit of the model using a confirmatory factor analysis with a randomly created test sample including 50% of the study households. The Comparative Fit Index [36], the Tucker Lewis Index [37], and the root mean square error of approximation [38] were used to assess the goodness of fit. The Comparative Fit Index and the Tucker Lewis Index assess the improvement of the model’s fit compared to a baseline model which has zero correlation between the observed variables. Values of 0.9 or larger indicate a good fit. The root mean square error of approximation ranges from 0 to 1, with values between 0.05 and 0.08 suggesting a good model fit. We used all three measures mentioned in the literature because there is no consensus of which is the favored one. For all statistical analyses SPSS version 23 was used. For the statistical tests we considered *p*-values <0.05 as statistically significant.

## Results

Table 2 presents the definition and descriptive statistics of household characteristics and of attitudes of the person mainly responsible for the food purchases in the household.

**Table 2:** Descriptive statistics of household characteristics and attitudes of the main shopper in the household (*n* = 13,125)

	Median/%	Interquartile range (iqr)
*Household characteristics*		
Household income: net monthly income reported in 17 income intervals. The mean of each interval was chosen as the income of the respective household (median, iqr, *n* = 13,125)	2625	1750
Number of persons in the household (median, iqr, n = 13,125)	2	1
Share of households with children aged 0 to 6 (%, n = 13,125)	13.0	
Share of households with children aged 7 to 13 (%, n = 13,125)	17.0	
Share of households with children aged 14 to 17 (%, n = 13,125)	6.0	
Lower education: share of households with a principal wage earner who has finished 9 years of elementary school but does not have additional professional training (%, n = 13,125)	26.4	
Higher education: share of households with a principal wage earner who has university degree (%, n = 13,125)	24.8	
Age: age of the person mainly responsible for the food purchases in the household reported in 11 age intervals. The mean of each interval was chosen as the relevant age of the respective person (median, iqr, n = 13,106)	52	25
*Attitudes of the person in the household mainly responsible for the food purchases*		
High price awareness: share of persons who reported a high or very high price awareness (%, n = 13,125)	37.6	
Paying attention to fat: share of persons who stated to pay high or very high attention to the fat content of foods (%, *n* = 11,660)	49.8	
Paying attention to sugar: share of persons who stated to pay high or very high attention to the sugar content of foods (%, n = 11,656)	35.2	
Paying attention to salt: share of persons who stated to pay high or very high attention to the salt content of foods (%, n = 11,656)	25.8	
Eating a low-carbohydrate diet: share of persons who stated that household members eat often or very often a low-carbohydrate diet (%, n = 11,633)	17.0	
Using supplements: share of persons who stated that household members use often or very often vitamin or mineral supplements (%, n = 11,660)	19.5	
Buying fortified foods: share of persons who stated to buy often or very often fortified foods (%, n = 11,656)	16.7	

### Identification of purchase patterns

Table 3 presents the results of the factor analysis. It is shown that three major factors (purchase patterns) were identified and these explained 52.7% of total variance in food purchases. The Cronbach’s Alpha, values of higher than 0.7 indicated good internal validities of the factors. The results of the confirmatory factor analysis showed a good model fit: All results of the Comparative Fit Index and the Tucker-Lewis-Index were greater than the cut-off value of 0.9 in all patterns. In contrast, the root mean square error of approximation indicated a mediocre to bad fit. However, it has been shown that this index tends to favor models including a larger number of variables over simple models which might explain the lower test statistics [39]. The communalities showed a good explanation of the variables in the model. For all food groups commonly bought by households the model explained a high proportion of the variance, for food groups that are bought by only a small proportion of households or are consumed less frequently, the communalities were lower.

**Table 3:** Factor loadings for food groups of three identified purchase patterns

	Factor loadings			
	Factor 1	Factor 2	Factor 3	Commu-
Food groups of German Food Guide Pyramid	natural	processed	traditional	nalities
*Plant based foods*				
Vegetables, salads, vegetable juices	**0.694**	0.372	0.326	0.727
Fruits, fruit juices	**0.748**	0.256	0.067	0.630
Whole grain products, nuts	**0.629**	0.253	−0.127	0.475
Potatoes	**0.541**	0.319	**0.425**	0.575
Refined grain products	0.303	**0.686**	0.358	0.691
Sugar, cakes, sweets, snacks, fatty potato prod.	0.379	**0.651**	0.326	0.673
*Animal based food*s				
Low fat meat (−products)	0.256	0.347	**0.555**	0.494
Fish	**0.604**	0.162	0.284	0.472
Low fat milk (−products)	0.173	**0.768**	−0.167	0.648
Meat (moderate fat content)	0.328	**0.436**	**0.646**	0.715
Milk (−products) (moderate fat content)	**0.484**	0.198	0.258	0.340
High fat meat products (sausages), eggs, cream	**0.423**	**0.471**	**0.588**	0.747
Bacon	0.096	0.041	**0.635**	0.414
*Fats and oils*				
Rape oil, walnut oil	0.115	0.368	0.214	0.195
Wheat germ oil, soybean oil	**0.568**	0.026	0.097	0.333
Margarine, corn oil, sunflower oil	0.109	**0.632**	**0.428**	0.595
Butter	**0.513**	0.057	**0.405**	0.431
Lard, solid vegetable fats	0.051	0.094	**0.570**	0.336
*Cronbach’s alpha*	*0.770*	*0.759*	*0.758*	
*Comparative Fit Index*	*0.939*	*0.949*	*0.953*	
*Tucker-Lewis-Index*	*0.918*	*0.915*	*0.935*	
*Root mean square error of approximation*	*0.086*	*0.129*	*0.048*	
*Explained variance (%)*	*39.894*	*7.046*	*5.792*	
*In total (%)*	*52.733*			

Factor loadings with absolute values >0.4 are marked in bold

As suggested in previous studies factor loadings larger than or equal to 0.4 were considered significant [13, 30]. The first of the three patterns listed in Table 3 was named ‘natural’ because it was characterized by higher loadings for vegetables, fruits, whole grain products, and potatoes in the plant-based food group. In the animal based food group, the pattern was more highly correlated with fish and milk and meat products with a higher fat content. Looking at the fats and oils group, the pattern was highly correlated with oils and butter. Overall, the first pattern was characterized by a combination of mainly healthy foods that are natural and unprocessed. In contrast, the second pattern was named ‘processed’ because it mainly combined foods that are industrially processed, namely refined grain products, sweets and snacks, low fat milk products, and margarine. The third factor was named ‘traditional’, because it showed higher loadings for all kinds of meat and, moreover, in the plant-based food group higher loadings appeared predominantly for potatoes. In the fats and oils group the less healthy variants had higher factor loadings.

### Analysis of diet quality

Mean values and standard deviations of different nutrients (e. g. μg/100 g) and energy densities (kcal/100 g) per quintile 1, 3 and 5 for every purchase pattern is shown in Table 4. Households with increasing quintiles in the natural pattern had higher densities for nearly all listed micronutrients. The related *p*-values indicate that this result is in most cases statistically significant at the 0.01 level. The only nutrient densities that did not reach statistical significance were those for calcium and fluoride.

**Table 4:** Nutrient and energy densities by quintiles of dietary patterns scores (mean values and (standard deviations))

	Quintiles of the natural food pattern				Quintiles of the processed food pattern				Quintiles of the traditional food pattern			
	Q1 lowest	Q3 medium	Q5 highest	sig. level for trend	Q1 lowest	Q3 medium	Q5 highest	sig. level for trend	Q1 lowest	Q3 medium	Q5 highest	sig. level for trend
*Micronutrients*												
Vitamin D^a^	0.015	0.017	0.020	<0.001	0.020	0.017	0.015	<0.001	0.016	0.018	0.018	<0.001
	(0.001)	(0.008)	(0.001)		(0.017)	(0.008)	(0.006)		(0.009)	(0.009)	(0.008)	
Vitamin E^b^	4.840	5.082	5.374	<0.001	5.189	5.125	4.946	<0.001	5.281	5.092	5.016	<0.001
	(2.958)	(1.619)	(1.409)		(2.492)	(1.831)	(1.691)		(1.888)	(1.789)	(1.629)	
Folic acid^b^	0.089	0.099	0.114	<0.001	0.108	0.099	0.093	<0.001	0.116	0.097	0.092	<0.001
	(0.049)	(0.025)	(0.027)		(0.053)	(0.026)	(0.021)		(0.034)	(0.025)	(0.021)	
Vitamin B12^a, h^	0.029	0.028	0.029	<0.01	0.032	0.028	0.027	<0.001	0.026	0.028	0.031	<0.001
	(0.018)	(0.001)	(0.016)		(0.022)	(0.011)	(0.007)		(0.009)	(0.001)	(0.012)	
Vitamin C^b^	31.969	39.882	51.681	<0.001	47.640	40.736	35.255	<0.001	48.100	39.507	37.868	<0.001
	(43.288)	(17.614)	(20.901)		(46.960)	(18.506)	(14.056)		(26.651)	(18.322)	(14.170)	
Calcium^c^	0.388	0.395	0.395	0.156	0.392	0.385	0.401	<0.05	0.486	0.380	0.330	<0.001
	(0.184)	(0.116)	(0.096)		(0.169)	(0.111)	(0.111)		(0.142)	(0.096)	(0.074)	
Magnesium^c^	0.131	0.136	0.144	<0.001	0.141	0.137	0.133	<0.001	0.154	0.134	0.127	<0.001
	(0.042)	(0.024)	(0.023)		(0.041)	(0.024)	(0.022)		(0.029)	(0.023)	(0.018)	
Iron^b^	6.163	6.644	6.838	<0.001	6.783	6.653	6.149	<0.001	6.684	6.562	6.479	<0.05
	(3.572)	(3.138)	(2.696)		(3.613)	(3.053)	(2.302)		(3.285)	(2.750)	(2.514)	
Iodine^b^	0.061	0.061	0.066	<0.001	0.064	0.061	0.062	0.123	0.063	0.061	0.065	<0.05
	(0.086)	(0.038)	(0.031)		(0.071)	(0.038)	(0.030)		(0.055)	(0.044)	(0.034)	
Fluoride^b^	0.722	0.668	0.715	0.946	0.703	0.680	0.675	0.253	0.608	0.685	0.794	<0.001
	(1.561)	(0.664)	(0.555)		(1.256)	(0.638)	(0.512)		(1.006)	(0.731)	(0.630)	
*Energy and macronutrients*												
Energy^d^	187.651	171.197	156.620	<0.001	170.658	171.609	171.624	0.562	151.949	174.740	178.371	<0.001
	(45.695)	(28.664)	(24.617)		(44.669)	(30.354)	(28.259)		(31.074)	(30.469)	(25.636)	
Sugar^e, i^	0.109	0.115	0.112	<0.01	0.101	0.117	0.120	<0.001	0.124	0.113	0.103	<0.001
	(0.060)	(0.039)	(0.034)		(0.055)	(0.041)	(0.035)		(0.043)	(0.040)	(0.032)	
Fat^f^	0.452	0.451	0.440	<0.001	0.463	0.448	0.436	<0.001	0.400	0.456	0.482	<0.001
	(0.095)	(0.060)	(0.057)		(0.094)	(0.059)	(0.055)		(0.058)	(0.057)	(0.051)	
P/S-quotient^g^	0.368	0.322	0.322	<0.001	0.308	0.333	0.355	<0.001	0.344	0.330	0.321	<0.001
	(0.262)	(0.104)	(0.108)		(0.249)	(0.122)	(0.110)		(0.143)	(0.112)	(0.098)	
Dietary Fiber ^c^	1127.042	1259.184	1385.179	<0001	1343.260	1281.378	1155.770	<0.001	1393.245	1246.668	1163.654	<0.001
	(909.131)	(477.388)	(408.422)		(918.051)	(491.448)	(393.141)		(556.211)	(513.354)	(371.663)	

^a^μg/1000 kcal, ^b^ μg/100 kcal, ^c^ mg/100 kcal, ^d^ kcal/100 g food, ^e^ percentage of calories from sugar (sucrose),^f^ percentage of calories from fat, ^g^ Ratio of polyunsaturated to saturated fatty acids, ^h^ Values for the five quintile groups in the natural pattern: 0.00285716, 0.00280611, 0.00283541, 0.00288286, 0.00293356, ^i^ Values for the five quintile groups in the natural pattern: 0.109, 0.114, 0.114, 0.114, 0.112

However, Pearson’s correlation results, shown in Table 5, indicated also for calcium a significantly positive value.

**Table 5:** Pearson’s correlations between purchase pattern scores and energy and nutrient densities

	Natural score		Processed score		Traditional score	
	coeff.	*p*-value	coeff.	*p*-value	coeff.	*p*-value
*Micronutrients*						
Vitamin D ^a^	0.168	<0.001	−0.150	<0.001	0.069	<0.001
Vitmain E ^a^	0.090	<0.001	−0.053	<0.001	−0.035	<0.001
Folic acid ^a^	0.278	<0.001	−0.143	<0.001	−0.210	<0.001
Vitamin B12 ^a^	0.032	<0.001	−0.116	<0.001	0.107	<0.001
Vitamin C ^a^	0.267	<0.001	−0.147	<0.001	−0.109	<0.001
Calcium^b^	0.025	<0.01	0.045	<0.001	−0.355	<0.001
Magnesium^b^	0.166	<0.001	−0.080	<0.001	−0.273	<0.001
Iron ^a^	0.070	<0.001	−0.085	<0.001	−0.028	<0.01
Iodine ^a^	0.029	<0.01	−0.016	0.061	0.026	<0.05
Fluoride ^a^	0.000	0.999	−0.018	<0.05	0.069	<0.001
*Energy and macronutrients*						
Energy^c^	−0.308	<0.001	0.007	0.442	0.202	<0.001
Sugar^d^	0.004	0.658	0.120	<0.001	−0.139	<0.001
Fat^e^	−0.076	<0.001	−0.140	<0.001	0.361	<0.001
P/S-quotient^f^	−0.073	<0.001	0.099	<0.001	−0.067	<0.001
Dietary fiber ^b^	0.149	<0.001	−0.109	<0.001	−0.121	<0.001

^a^μg/100 kcal, ^b^ mg/100 kcal, ^c^ kcal/100 g food, ^d^ percentage of calories from sugar (sucrose), ^e^ percentage of calories from fat, ^f^ PUFAs/SFAs

Both Tables 4 and 5 indicated an inverse association between the natural pattern score and the energy density, which means that a rising orientation towards this pattern was combined with a more favorable energy density. Among the macronutrients no significant correlation could be detected for sugar (sucrose). Both, the percentage of calories from fat and the p/s-quotient, which is the ratio of polyunsaturated to saturated fatty acids, were significantly negatively associated with this pattern, indicating a more favorable fat density but a less favorable fat composition. The density of dietary fiber showed a positive association with the natural score.

Households that were more oriented towards the processed food pattern showed negative associations with all micronutrient densities except for calcium for which the correlation was significantly positive (Table 5). The correlation coefficient between energy density and this pattern score was close to zero which corresponded to the mean values for energy density. These values were nearly the same in the three quintile groups (Table 4). The percentage of calories from sugar was clearly positively associated with the score. The percentage of calories from fat showed a significant negative and the p/s-quotient a significant positive association with the processed pattern score. That means, both evaluation criteria for fat consumption, density and composition, were more favorable with higher pattern scores. Dietary fiber showed a negative and thus less favorable association with this pattern.

Results regarding the association between the traditional pattern and the micronutrient densities were mixed: Whereas the densities for the vitamins D and B12 as well as for iodine and fluoride were significantly positively correlated with the pattern score, the remaining nutrient densities showed significant negative correlation coefficients (see Table 5). Referring to the energy and macronutrient densities, a more favorable association was only detected for sugar which showed a negative correlation with this pattern. All other criteria indicated a more unfavorable diet with higher pattern scores, with higher fat density and higher dietary fiber density.

### Associations between purchase pattern scores and socio-economic factors

**Fig. 2:**
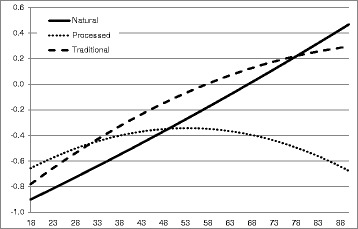
Association between age and food pattern scores

Table 6 lists the results of the regression analyses. The ANOVAs of all three models were statistically significant. As shown, income was highly significant for all three purchase patterns. However, whereas the natural pattern had a positive sign and was therefore consumed more as income increased, the other two patterns showed a negative sign. A larger household size was significantly and positively associated with all pattern scores. However, holding the household size constant, the number of children showed a negative association. Regarding the education level it is shown that at a higher level the natural pattern was more common whereas at a lower level the traditional pattern was more prevalent. The processed pattern showed no significant differences concerning education level. Age was significantly positively associated with all three purchase patterns, indicating increasing adherence with increasing age. However, the significance of the quadratic transformation suggests the relationship between age and purchase patterns to be non-linear in the processed and traditional pattern. From the parameter estimates, it could be derived that the processed score increased with rising age, reached its maximum when the person was 53 years and then decreased again. In contrast, the curve shape of the traditional pattern was slightly convex. These associations are illustrated in Fig. 2. The price level at which foods were purchased was higher in households with a stronger adherence to the natural and traditional pattern and lower in households where the processed pattern was more prevalent. Consistent with this, the households of the natural pattern stated to be less price conscious, whereas households of the processed pattern had higher price awareness. In contrast, households of the traditional pattern had the highest price level and also the highest price consciousness.

**Table 6:** The impact of socio-demographic and attitude variables on food pattern scores

	Natural pattern				Processed pattern				Traditional pattern			
	reg.coeff.	95% CI		*p*-value	reg.coeff.	95% CI		*p*-value	reg.coeff.	95% CI		*p*-value
Constant	−1.572	−1.801	−1.343	<0.001	−1.403	−1.626	−1.180	<0.001	−1.641	−1.878	−1.403	<0.001
Household income / 1000^a^	0.099	0.080	0.119	<0.001	−0.046	−0.066	−0.027	<0.001	−0.045	−0.065	−0.024	<0.001
Numb. pers. in household	0.198	0.171	0.225	<0.001	0.358	0.332	0.385	<0.001	0.352	0.323	0.380	<0.001
Numb. childr. Aged 0-6	−0.091	−0.144	−0.038	<0.01	−0.191	−0.243	−0.140	<0.001	−0.390	−0.445	−0.334	<0.001
Numb. childr. Aged 7-13	−0.129	−0.170	−0.087	<0.001	0.034	−0.007	0.075	0.100	−0.286	−0.329	−0.242	<0.001
Numb. childr. Aged 14-17	−0.126	−0.199	−0.053	<0.01	0.024	−0.047	0.095	0.502	−0.210	−0.286	−0.134	<0.001
Lower education	−0.093	−0.135	−0.051	<0.001	0.040	−0.001	0.081	0.058	0.200	0.156	0.243	<0.001
Higher education	0.191	0.147	0.234	<0.001	−0.010	−0.053	0.032	0.639	−0.173	−0.219	−0.128	<0.001
Age	0.016	0.007	0.025	<0.01	0.027	0.018	0.035	<0.001	0.031	0.021	0.040	<0.001
Age^2^/ 100^a^	0.003	−0.005	0.011	0.465	−0.025	−0.033	−0.017	<0.001	−0.015	−0.023	−0.006	<0.001
Level of prices	0.056	0.075	0.036	<0.001	−0.145	-0.126	-0.164	<0.001	0.069	0.089	0.049	<0.001
High price awareness	−0.042	−0.078	−0.005	<0.05	0.095	0.059	0.131	<0.001	0.106	0.068	0.144	<0.001
Paying attention to fat	0.070	0.032	0.109	<0.001	0.122	0.085	0.159	<0.001	−0.174	−0.214	−0.134	<0.001
Paying attention to sugar	0.184	0.144	0.224	<0.001	−0.046	−0.085	−0.007	<0.05	−0.047	−0.089	−0.005	<0.05
Paying attention to salt	0.011	−0.032	0.054	0.623	0.006	−0.036	0.048	0.782	−0.077	−0.122	−0.032	<0.001
Eating low carbohydr. Diet	−0.007	−0.057	0.043	0.781	−0.016	−0.065	0.033	0.520	−0.010	−0.062	0.042	0.715
Using supplements	0.051	0.006	0.095	<0.05	0.000	−0.044	0.044	0.999	−0.115	−0.161	−0.068	<0.001
Buying fortified foods	−0.151	−0.198	−0.103	<0.001	0.059	0.012	0.106	<0.05	0.036	−0.014	0.085	0.160
*R* ^*2*^ *(adjusted)*	*0.147*				*0.205*				*0.126*			

^a^ In order to indicate the effect of the independent variable without showing more decimal places, we divided the income and age values by 1000 and 100 respectively

### Associations between purchase pattern scores and attitudes

With regard to the attitude variables it is shown that households with a higher adherence to the natural pattern stated more often to pay attention to the food’s fat and sugar content and to use supplements. However, they more often reject buying fortified foods. Households of the processed pattern more often stated to pay attention to the fat but not to the sugar content. In addition, they buy fortified foods more often. Households with a higher adherence to the traditional pattern payed less often attention to any of the guidelines and, furthermore, stated less often to use supplements.

## Discussion

German Food Guide Pyramid: In many previous dietary pattern analyses, individual food items were grouped into common food groups in preparation for the factor analysis. In this analysis we used the classification scheme of the German Food Guide Pyramid to classify the 1954 individual food items into 18 groups. These groups are characterized by similar nutrient profiles considering criteria which value foods in an explicit and transparent manner according to health aspects. The suitability of the 18 food groups for this analysis was verified by the communalities which show the proportion of variation in each variable that is explained by the model. In our analysis 12 variables had communalities of 0.5 and above and especially the variations of foods from the animal and plant based food groups were well explained by the model.

### Food purchase patterns and diet quality

Using the factor analysis we identified three purchase patterns. Comparable patterns were determined in previous dietary pattern studies: the ‘natural’, which is similar to a healthy pattern in previous studies [15, 40], the ‘traditional’ [41, 42], and the ‘processed’ [15, 43]. However, whereas the preceding studies used food intake data this study referred to purchase data. In particular, the processed food pattern in this study differs from that identified in other studies. Here, the processed pattern was characterized by meat products richer in fat, sweets, snacks and cakes in combination with reduced fat dairy products. In previous studies, the reduced fat dairy products usually appeared in the healthy food pattern [14, 40].

Overall, the results indicated that none of the three purchase patterns identified in this study, complied fully with current food guidelines. However, among the three identified patterns, the natural one could be characterized as comparatively healthy. In accordance with current dietary guidelines, it contained vegetables, fruits, whole meal products and fish. This combination of foods was reflected in more favorable micronutrient, energy, fat, and fiber densities. However, instead of combining these foods with milk and meat products with a reduced fat content, as recommended in dietary guidelines [44, 45], those with higher fat contents were found. Furthermore, higher factor loadings occurred for butter instead of margarine. Compared with margarine, butter has, on average, a less favorable p/s- ratio and contains more energy which can be explained by the high number of fat reduced and fortified types of margarine on the German market. As a consequence a less favorable p/s –quotient was observed in this pattern. The association of the natural pattern score with sugar showed no clear tendency, indicating that a higher adherence was not necessarily related with reduced sugar purchasing. In the processed pattern, higher purchases of margarine and plant oils as well as low fat milk and milk-products were found. These foods were combined with refined grain products, sweets, and high fat meat and meat-products all of which should be consumed moderately. As a consequence, nearly all individual micronutrient densities declined with higher scores. Because of the lower purchases of fruits, vegetables and whole grain products in this pattern a reduced fiber density could be observed. A further drawback of this pattern was the increased sugar density reflecting higher factor loadings for the sweets and snacks group. However, despite of an increased purchasing of these foods, the energy density remained constant among score quintiles. An explanation could be that the higher purchases of sweets and snacks were compensated by fat reduced variants of dairy products and of margarine instead of butter. The purchases of fat reduced products were reflected in a reduced fat density and in a more favorable fat composition. The traditional pattern had high factor loadings particularly for all kinds of meat and for fats with a higher share of saturated fatty acids, and therefore was to be regarded as unfavorable. This purchase behavior was reflected in decreasing micronutrient and dietary fiber densities, increasing fat and energy densities and an unfavorable fat composition. Such associations between the traditional pattern and diet quality criteria have frequently been detected in previous studies [9, 42]. The only criteria that could be evaluated positively in this pattern, was the percentage of calories from sugar which was inversely correlated with the pattern score. This observation was consistent with lower purchases of the sweets and snacks category. However, overall the diet quality of the traditional pattern could be assessed as unfavorable.

### Association with socio-economic factors

To identify the household groups with their specific needs of improvement of diet quality, associations with socio-economic characteristics and attitudes were investigated. Households with a higher adherence to the natural pattern had a higher income, a higher education level, and they purchased foods at a higher price level. In contrast, households with a stronger preference for the traditional pattern had a lower income and a lower education level. These results were consistent with several studies that found an association between diet quality and socio-economic status [30, 46, 47]. Households with a stronger preference for the processed pattern had a lower income and purchased their foods at a lower price level. However, it showed no significant association with education and thus was common in all education levels. Regarding age, the findings indicated that the adherence to a pattern was generally more pronounced when the person responsible for the food purchases was older. This relationship was valid for the entire age range in the natural and traditional pattern and, until the age of 53, in the processed pattern. Whereas household size was positively associated with the adherence to a food pattern in all three pattern groups, the presence of children showed a negative association. This implies that the presence of adults determined a stronger adherence to a pattern. Taking the factors age and the presence of adults and children into account, it can be assumed, that purchase patterns generally seemed to be more pronounced when the household consisted of older people. This could support a finding of a previous study detecting that dietary habits are mainly formed at a younger age and then often remain stable [48]. However, further analyses are necessary to confirm our assumption.

### Association with attitudes

The results on the associations between purchase pattern scores and attitude variables were largely as expected: Households with a higher adherence to the natural pattern stated to pay attention to dietary guidelines and to use supplements. However, given that these households seem to pay attention to a food’s sugar content it could have been expected that the percentage of sugar decreased with a rising pattern score. In fact, no clear tendency could be observed. In contrast, households of the traditional pattern do not seem to pay attention to sugar, but actually had the lowest percentage of sugar in their food composition. One explanation why households of the natural pattern had relatively high sugar content in their food basket could be that they buy sugared products rather than products with artificial sweeteners. However, further research is necessary to find out more about the association between attitudes and behavior in the purchasing of sugar. The households with a higher adherence to the processed pattern stated to pay attention to a food’s fat but not to the sugar content and both statements were consistent with their behavior. Overall, it can be summarized that attitudes and actual behavior were largely consistent.

### Database

Whereas previous studies used individual food intake data to derive dietary patterns this study made use of household purchase data to identify food purchase patterns. It is important to note that, because of the different data, the results might not be completely comparable. Both data have different advantages and disadvantages which are reflected in the identified patterns.

In the purchase data used here households are not directly asked about their dietary habits, as is the case with intake data. Therefore, it can be assumed that they are less biased by systematic over- and underreporting. In addition, the detailed documentation of households’ food purchases allowed a precise assignment of each product to the food categorization of the German Food Pyramid. For a precise assignment the distinction between the foods’ fat contents, among others, was essential. Such a distinction has been made in very few previous dietary pattern studies: some of them considered fat contents of dairy products [43, 49, 50], others referred to different types of meat such as poultry, beef and pork [41, 42, 51], but did not consider fat reduced variants within the types of meat. None of them considered fat reduced variants across all products which is important to identify respective patterns.

However, the used data also have some limitations: Firstly, out of home purchases are not included and therefore the food purchase patterns and its diet quality exclusively refers to the in home consumption. However, this part accounts for 77% of all food expenses in Germany [52] and as foods purchased out of home are more expensive than those purchased for in home consumption, the quantitative food purchases here are higher than 77%.

Secondly, food purchases differ from food intakes because of waste. A UK study found that approximately 20% of all purchases were thrown away by the households [53]. The most prominent food group by weight was fresh vegetables and salad, which made up 23%, followed by beverages (16%) and fresh fruits (13%). Hence, making comparisons with food intake means that in particular vegetables, salads and fruits would be overrepresented within the identified patterns (beverages were not included in our study). With respect to the nutrients it is to be noted that, when linking the purchases with the nutrient database, we took the respective variant ‘with’ or ‘without inedible (unavoidable) kitchen waste’. Therefore, the difference between purchased and actually consumed nutrients refers to the part of waste that could be avoided by the households.

Thirdly, the data display the food purchase habits and its dietary quality with regard to the whole household. With intake data, individual dietary habits could be displayed in its pure form, with household data instead, the dietary habits of all household members on average is represented. Nevertheless, as we were able to identify clearly definable purchase patterns, consumption habits of household members seemed not to be too far away from each other. However, since individual household members could have different consumption habits, the results should not be used for drawing conclusions for individuals.

A further point worth noting with regard to the data is that the manual scanning of products is very labor intensive and therefore, it is conceivable that some purchases were left out by the households. However, when the number of scanned products significantly decreases during the data collection period, the households are excluded from the survey by the GFK. Furthermore, it should be mentioned that the data collection was in 2011. As dietary habits have not changed significantly in recent years [1, 54] it can be assumed that the derived patterns are still valid. Not least it should be mentioned that, although the linkage of the purchase data with the nutrient composition database resulted in a reduction of the number of products, a significant impact on the results are not to be expected. The main reason for the reduction was an aggregation of the very detailed fat and flavor groups to broader groups. These broader groups are sufficient for a precise assignment to the food groups of the German Food Pyramid which served as the basis for the factor analysis.

To conclude, in order to address the limitations associated with household purchase data, it would be desirable to conduct a comparable study using individual food intake data. Combining the results of both kinds of analysis could help to get deeper insights into population’s dietary habits.

## Conclusions

One main result of this study was that none of the detected purchase patterns complied fully with recommendations given from nutritionists. In each pattern there were specific aspects that could be improved, whereas others were in line with actual dietary guidelines. This result suggests that public health measures should focus more on information about necessary improvements for individual food patterns. It can be expected that measures focusing on specific aspects of dietary quality for individual patterns are more promising than giving solely general dietary recommendations. However, a major challenge is to identify the population groups with their specific needs of improvement and moreover, to design respective measures. This analysis provided some indications for pattern specific dietary issues and characteristics associated with the pattern groups: Because of its unfavorable fat composition households with a higher adherence to a natural food pattern should particularly be informed about the association between the consumption of high-fat dairy products and the p/s-quotient and, moreover, how this ratio could affect human health. As the adherence to this pattern was associated with a higher socio-economic status such measures should particularly be targeted at this group. Households with a higher adherence to the other two patterns, who had a lower socio-economic status, had a relatively low purchasing of healthier foods (e.g., vegetables and fruits) and hence their micronutrient and fiber densities were lower. Instead, they had higher purchases of sugar sweetened foods (processed pattern) and of foods with less favorable fat contents (traditional pattern). However, as these households were aware of their specific advantages and disadvantages of their diet quality they should be informed about the necessity to pay attention to all criteria affecting diet quality and health. This could be carried out in the form of school education programs especially directed to low socio-economic status groups.
